# Early achievement of deep remission predicts low incidence of renal flare in lupus nephritis class III or IV

**DOI:** 10.1186/s13075-018-1576-1

**Published:** 2018-05-02

**Authors:** Hironari Hanaoka, Harunobu Iida, Tomofumi Kiyokawa, Yukiko Takakuwa, Kimito Kawahata

**Affiliations:** Division of Rheumatology and Allergology, Department of Internal Medicine, St. Marianna University School of Medicine, Kanagawa, 216-8511 Japan

## ᅟ

Recommendations for lupus nephritis (LN) management specify that the therapeutic target should be a complete renal response (CR) [1], defined as a urine protein:Cr ratio (UPCR) of 0.5 g/gCr (50 mg/mmol) and normal or near-normal renal function. Earlier studies suggested that patients who achieved CR experienced fewer renal flares than those who achieved partial remission, defined as a 50% reduction of proteinuria [2]. Among patients who achieved CR (less than 0.50 g/gCr of UPCR), however, the renal outcome of those who achieved a value below the normal UPCR limit of 0.15 g/gCr was unclear. We recently reported that an early renal response may predict a good renal or systemic outcome [3, 4]. In this study, we investigated whether it is beneficial to achieve deep remission early by evaluating flare rate, Systemic Lupus International Collaborating Clinics/American College of Rheumatology Damage Index (SDI), cumulative glucocorticoid dose, and eGFR level.

We retrospectively assessed 69 patients with biopsy-proven LN class III or IV who achieved CR in our hospital. We divided them into two groups based on whether deep remission was achieved, defined as less than 0.15 g/gCr UPCR, and compared cumulative flare rates [1], defined as estimated glomerular filtration rate (eGFR) decreasing by ≥ 10%, active urine sediment, or increasing UPCR > 1.0 g/gCr after achieving CR. Furthermore, we analyzed the additional effect of early achievement of CR, defined as CR within 3 months after induction therapy. Clinical characteristics between the two groups were compared using the non-parametric Mann-Whitney *U*-test. Frequencies of clinicopathological characteristics were compared using the Chi-square test. Cumulative flare free rates were calculated using the Kaplan-Meier method, and differences between the two groups were tested with a log-rank test. To identify independent parameters that predict CR at 3 years, we performed multivariate analysis.

During the 3-year period, 55 of 69 CR patients achieved deep remission while 14 did not. Among clinical features at baseline, the proportion of females was significantly higher among patients with deep remission (*p* = 0.01; Table 1).

**Table 1:** Baseline clinical and renal pathological features of LN patients with or without deep remission

Baseline characteristics	Deep remission		*p*
	Achieved (*n* = 55)	Not achieved (*n* = 14)	
Sex (percentage female)	48 (87.3)	9 (64.2)	0.01
Age (years)	39.1 ± 12.4	39.9 ± 10.7	0.3
BMI (kg/m^2^)	22.4 ± 3.3	20.9 ± 2.3	0.5
Systolic blood pressure (mmHg)	127.8 ± 17.0	136.4 ± 21.5	0.2
Diastolic blood pressure	79.7 ± 13.6	83.6 ± 14.5	0.3
Disease duration (years)	5.1 ± 6.8	8.0 ± 6.4	0.4
SLEDAI	15.6 ± 4.8	13.0 ± 4.8	0.4
SDI	0.4 ± 0.6	0.6 ± 0.8	0.7
Proteinuria (g/gCr)	2.7 ± 2.1	3.7 ± 1.8	0.07
eGFR (mL/min)	76.6 ± 28.2	72.2 ± 32.8	0.9
Anti-dsDNA antibody (IU/mL)	177 ± 274	112 ± 116	0.6
Anti-cardiolipin antibody (IU/mL)	21.8 ± 30.7	14.7 ± 28.5	0.5
Lupus anticoagulant-positive (%)	3 (5.5)	1 (7.1)	0.6
CH50 (U/ml)	17.1 ± 9.3	24.4 ± 14.2	0.06
Prednisolone (mg/day)	45.5 ± 15.1	37.1 ± 10.1	0.08
Induction therapy			
IVCY (%)	29 (52.7)	5 (35.7)	0.4
MMF (%)	8 (14.5)	2 (14.3)	0.9
Tacrolimus (%)	8 (14.5)	2 (14.3)	0.9
PSL monotherapy (%)	6 (10.9)	2 (14.3)	0.8
Others (%)	4 (7.3)	3 (21.4)	0.1
Renal pathological findings			
ISN/RPS classification			
III or III + V (%)	26 (47.3)	5 (35.7)	0.4
IV or IV + V (%)	29 (52.7)	9 (64.3)	0.4
Endocapillary hypercellularity (%)	39.2 ± 18.6	46.0 ± 30.2	0.5
Leukocyte infiltration (%)	2.0 ± 4.9	2.2 ± 4.1	0.4
Subendothelial hyaline deposits (%)	29.1 ± 30.2	30.1 ± 28.9	0.3
Fibrinoid necrosis/karyorrhexis (%)	7.0 ± 11.1	8.1 ± 18.1	0.3
Cellular crescents (%)	7.0 ± 11.1	8.1 ± 18.1	0.7
Interstitial inflammation (%)	2.1 ± 3.7	2.3 ± 7.4	0.5
Glomerular sclerosis (%)	2.5 ± 7.1	3.9 ± 8.2	0.4
Fibrous crescents (%)	2.1 ± 2.0	2.2 ± 3.1	0.7
Tubular atrophy (%)	2.8 ± 4.7	3.3 ± 5.9	0.5
Interstitial fibrosis (%)	4.7 ± 6.8	5.1 ± 5.9	0.6
Activity index	5.2 ± 3.0	5.1 ± 3.8	0.5
Chronicity index	1.4 ± 0.4	1.4 ± 1.3	0.7

*dsDNA* double-stranded DNA, *IVCY* intravenous cyclophosphamide, *MMF* mycophenolate mofetil, *SDI* Systemic Lupus International Collaborating Clinics/American College of Rheumatology Damage Index, *SLEDAI* Systemic Lupus Erythematosus Disease Activity Index

**Fig. 1:**
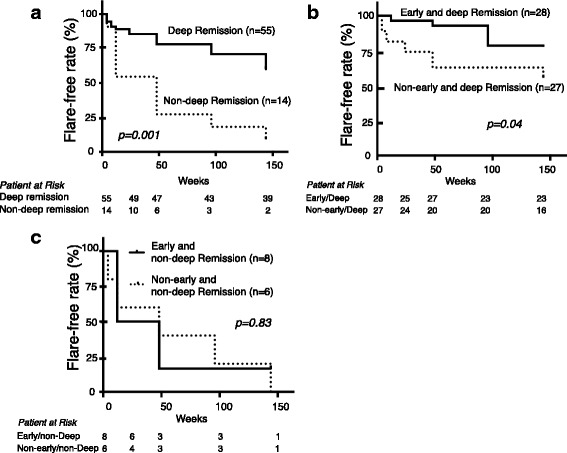
Cumulative renal relapse-free rate in the 3 years after induction therapy. Comparison of relapse-free rate between patients with deep remission and those without (**a**). Comparison of relapse-free rate between patients with early remission and those without among patients who had achieved deep remission (**b**) and non-deep remission (**c**)

**Fig. 2:**
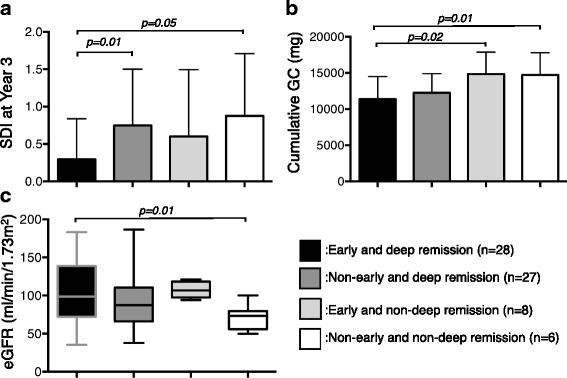
Comparison of SDI, glucocorticoid dose, and eGFR level at year 3. Patients were divided into four groups depending on achievement of deep remission and early CR and SDI (**a**), glucocorticoid dose (**b**), and eGFR level (**c**) were compared among them. *SDI* Systemic Lupus International Collaborating Clinics/American College of Rheumatology Damage Index, *GC* glucocorticoid, *eGFR* estimated glomerular filtration rate. The error bars represent mean ± SD

We found a significantly higher flare-free rate among patients who achieved deep remission compared with those who did not (*p* = 0.001; Fig. 1a). For patients with deep remission, those with early CR had a higher flare-free rate than those without (*p* = 0.04) (Fig. 1b), but significant difference was found in those with non-deep remission (Fig. 1c). Multivariate analysis to predict sustained CR indicated that early achievement of deep remission was an independent factor (odds ratio 3.62, 95% confidence interval 1.1–18.9, *p* = 0.05). Regarding SDI, cumulative glucocorticoid dose, and eGFR level at year 3, patients with early deep remission had the most favorable result compared to the other groups (Fig. 2).

In this study, we found that achieving early and deep remission predicts a good renal outcome in patients with LN class III or IV. Since renal flare predicts a worse prognosis [5], determining the method of treatment to ensure long-term maintenance of CR is challenging. Our results suggest that deep remission might be a more beneficial therapeutic goal than that of the EULAR/ERA-EDTA recommendations regarding the prevention of renal flare. A future multi-center, prospective study is required to confirm our findings.

## Abbreviations

CR: Complete renal response
EULAR/ERA-EDTA: The Joint European League Against Rheumatism and European Renal Association–European Dialysis and Transplant Association
LN: Lupus nephritis
SDI: Systemic Lupus International Collaborating Clinics/American College of Rheumatology Damage Index
UPCR: Urine protein:Cr ratio
